# BeiDou Inter-Satellite-Type Bias Evaluation and Calibration for Mixed Receiver Attitude Determination

**DOI:** 10.3390/s130709435

**Published:** 2013-07-22

**Authors:** Nandakumaran Nadarajah, Peter J. G. Teunissen, Noor Raziq

**Affiliations:** 1 GNSS Research Centre, Department of Spatial Sciences, Curtin University, GPO Box U1987, Perth, WA 6845, Australia; E-Mails: p.teunissen@curtin.edu.au (P.J.G.T.); noor.raziq@curtin.edu.au (N.R.); 2 Delft Institute of Earth Observation and Space Systems (DEOS), Delft University of Technology, PO Box 5058, 2600 GB Delft, The Netherlands

**Keywords:** Global Navigation Satellite Systems (GNSS), BeiDou system (BDS), inter-satellite-type biases, attitude determination, multivariate constrained integer least-squares(MC-LAMBDA), carrier phase ambiguity resolution

## Abstract

The Chinese BeiDou system (BDS), having different types of satellites, is an important addition to the ever growing system of Global Navigation Satellite Systems (GNSS). It consists of Geostationary Earth Orbit (GEO) satellites, Inclined Geosynchronous Satellite Orbit (IGSO) satellites and Medium Earth Orbit (MEO) satellites. This paper investigates the receiver-dependent bias between these satellite types, for which we coined the name “inter-satellite-type bias” (ISTB), and its impact on mixed receiver attitude determination. Assuming different receiver types may have different delays/biases for different satellite types, we model the differential ISTBs among three BeiDou satellite types and investigate their existence and their impact on mixed receiver attitude determination. Our analyses using the real data sets from Curtin's GNSS array consisting of different types of BeiDou enabled receivers and series of zero-baseline experiments with BeiDou-enabled receivers reveal the existence of non-zero ISTBs between different BeiDou satellite types. We then analyse the impact of these biases on BeiDou-only attitude determination using the constrained (C-)LAMBDA method, which exploits the knowledge of baseline length. Results demonstrate that these biases could seriously affect the integer ambiguity resolution for attitude determination using mixed receiver types and that *a priori* correction of these biases will dramatically improve the success rate.

## 1. Introduction

The Chinese BeiDou System (BDS), having different types of satellites, is an important addition to the ever growing system of Global Navigation Satellite Systems (GNSS). The BDS currently under development will consist of five Geostationary Earth Orbit (GEO) satellites, three Inclined Geosynchronous Satellite Orbit (IGSO) satellites and twenty-seven Medium Earth Orbit (MEO) satellites [1,2]. Presently, it consists of five GEO, five IGSO and four MEO satellites transmitting navigation signals in quadrature phase-shift keying (QPSK) modulation on a total of three frequency bands (B1, B2, B3). This paper investigates the receiver-dependent bias between these satellite types, for which we coined the name “inter-satellite-type bias” (ISTB), and its impact on mixed receiver attitude determination.

Having 14 fully operational satellites, BDS has already had the standalone capability of satellite-based positioning, navigation and timing (PNT) solutions, at least in the Asia-Pacific region. Analyses of BeiDou-based PNT solutions have been reported in various studies. Apart from simulation-based studies in [3–7], analyses of BeiDou PNT with real data have been reported in [8–18]. Measurement quality and relative positioning analyses with real data collected using Chinese GNSS receivers (UB240-CORS) are reported in [10,19]. Precise point positioning results with the same receiver type are reported in [15]. Initial assessment of real data collected using non-Chinese GNSS receivers and with post-processed orbit and clock products [20,21] independent of the control segment is reported in [8]. The same products are used to analyse precise point positioning in [11,14] and triple-frequency relative positioning in [9]. Similar products are used in [12] to analyse the contribution of BeiDou in single point positioning. With the recent release of BeiDou interface control document (ICD) [1], one can expect increased research on BeiDou based-PNT solutions outside China.

Multiple GNSS receivers/antennas rigidly mounted on a platform can be used to determine platform attitude (orientation) (see, for example [22–28]). GNSS-based attitude determination offers several advantages, including that it is not affected by drift, is lower in cost and requires less maintenance than traditional methods. Precise attitude determination, however, relies on successful resolution of the integer carrier phase ambiguities. The least squares ambiguity decorrelation adjustment (LAMBDA) method [29] is currently the standard method for solving unconstrained and linearly-constrained GNSS ambiguity resolution problems (see, for example [30–37]). For such models, the method is known to be numerically efficient and optimal in the sense that it provides integer ambiguity solutions with the highest possible success-rate [38–40]. To exploit the known baseline length, we make use of the constrained (C-)LAMBDA method [41–49]. BeiDou-based attitude determination using identical receivers has been analysed in [16–18].

In this paper, we consider *mixed receiver* attitude determination using single- and dual-frequency BeiDou observables. Assuming different receiver types may have different delays/biases for different satellite types, we model the differential ISTBs among three BeiDou satellite types. We develop an extended GNSS model, taking into account these biases, and describe the estimation of these biases. Our analyses using the data from two real data campaigns (one is with Curtin's permanent GNSS array consisting of different types of BeiDou-enabled receivers, and the other is with zero-baseline using BeiDou enabled receivers) reveal the existence of non-zero ISTBs between different BeiDou satellite types. We observe that these biases are stable and constant. Hence, we use *a priori* estimated biases to correct BeiDou observations, so that one can use classical double difference processing without loosing redundancy. We then analyse the impact of these biases on BeiDou-only attitude determination using the constrained (C-)LAMBDA method, which exploits the knowledge of baseline length. Results demonstrate that ISTBs could seriously affect the integer ambiguity resolution for attitude determination using mixed receiver types and that *a priori* correction of these biases will dramatically improve the success rate.

This contribution is organized as follows. Section 2 describes the functional and stochastic model for BeiDou observations, with special attention to ISTBs and associated processing approaches. Section 3 formulates the quadratically-constrained BeiDou model for attitude determination and describes the C-LAMBDA method. Section 4 demonstrates the existence of non-zero ISTBs between different BeiDou satellite types using real data and presents the results of attitude determination, revealing the impact of ISTBs. Finally, Section 5 contains the summary and conclusions of this contribution.

## 2. BeiDou Observations

This section presents the BeiDou observation model, distinguishing satellite types, namely GEO, IGSO and MEO, to accommodate receiver-dependent delays (biases) for different satellite types. Since the BeiDou system uses the code division multiple access (CDMA) technique, the code and phase observations of receiver **r** tracking satellite *s_β_* of type *β* at frequency *j*, denoted by $p_{r,j}^{s_{\beta }}$ and $\phi_{r,j}^{s_{\beta }}$, respectively, are given as [50]:

(1) $$p_{r,j}^{s_{\beta }}=p_{r}^{s_{\beta }}+dt_{r}-dt^{s_{\beta }}+a_{r,j}^{s_{\beta }}+d_{r,j}^{,\beta }-d_{,j}^{s_{\beta }}+e_{r,j}^{s_{\beta }},\;j=1,\ldots ,f;\;s_{\beta }=1,\ldots ,m_{\beta };\;\beta =1,\ldots ,\eta$$

(2) $$\phi_{r,j}^{s_{\beta }}=p_{r}^{s_{\beta }}+dt_{r}-dt^{s_{\beta }}+\alpha_{r,j}^{s_{\beta }}+\lambda_{j}(\delta_{r,j}^{,\beta }+\varphi_{r,j}-\delta_{,j}^{s_{\beta }}-\varphi_{,j}^{s_{\beta }}+N_{r,j}^{s_{\beta }})+\in_{r,j}^{s_{\beta }}$$

with *f* the number of frequencies, *m_β_* the number of type-*β* satellites tracked, *η* the number of satellite types, $p_{r}^{s_{\beta }}$ the topocentric distance between receiver *r* and satellite *s_β_*, *dt_r_* the receiver clock error, *dt^s_β_^* the satellite clock error, $a_{r,j}^{s_{\beta }}$ the (frequency-dependent) code atmospheric delay, $\alpha_{r,j}^{s_{\beta }}$ the (frequency-dependent) phase atmospheric delay, $d_{r,j}^{,\beta }$ the (satellite-type-dependent) hardware code delay in the receiver, $d_{,j}^{s_{\beta }}$ the hardware code delay in the satellite, *λ_j_* the wavelength, $\delta_{r,j}^{,\beta }$ the (satellite-type-dependent) hardware phase delay in the receiver, *φ_r,j_* the initial phase in the receiver, $\delta_{,j}^{s_{\beta }}$ the hardware phase delay in the satellite, $\varphi_{,j}^{s_{\beta }}$ the initial phase in the satellite, $N_{r,j}^{s_{\beta }}$ the (integer) phase ambiguities, $e_{r,j}^{s_{\beta }}$ all other code errors, including measurement noise, and $\in_{r,j}^{s_{\beta }}$ all other phase errors, including measurement noise. For simplicity of formulation, we assume that satellites are ordered, such that the first *m*_1_ satellites are of type 1, the next *m*_2_ satellites are of type 2, and so on ( $\sum_{\beta =1}^{\eta }m_{\beta }=m$, the number of tracked satellites). Note that all variables are expressed in meters; except the satellite-type-dependent biases of phase observations and the ambiguity, which are expressed in cycles.

The between-receiver single difference (SD) pseudo-range and carrier-phase observations of two BeiDou receivers, *r* and 1, at frequency *j* from satellite *s_β_* of type *β*, denoted as $p_{1r}^{s_{\beta }}$ and $p_{1r}^{s_{\beta }}$, respectively, are given as:

(3) $$p_{1r,j}^{s_{\beta }}=p_{r,j}^{s_{\beta }}-p_{1,j}^{s_{\beta }}=p_{1r}^{s_{\beta }}+dt_{1r}+a_{1r,j}^{s_{\beta }}+d_{1r,j}^{,\beta }+e_{1r,j}^{s_{\beta }}$$

(4) $$\phi_{1r,j}^{s_{\beta }}=\phi_{r,j}^{s_{\beta }}-\phi_{1,j}^{s_{\beta }}=p_{1r}^{s_{\beta }}+dt_{1r}+\alpha_{1r,j}^{s_{\beta }}+\lambda_{j}(\delta_{1r,j}^{,\beta }+\varphi_{1r,j}+N_{1r,j}^{s_{\beta }})+\in_{1r,j}^{s_{\beta }}$$

where the satellite-specific biases are eliminated. Attitude determination for a small platform considered in this paper is based on multiple BeiDou receivers forming very short-baselines for which the differential atmospheric delays can be ignored, that is, $a_{1r,j}^{s_{\beta }}=\alpha_{1r,j}^{s_{\beta }}=0\forall j,s_{\beta }$

Further differencing the above SD observables between satellites eliminates the receiver-dependent biases: with the first satellite of the satellite-type 1 as the pivot, the double difference (DD) observables are given as:

(5) $$p_{1r,j}^{1_{1}s_{\beta }}=p_{1r,j}^{s_{\beta }}-p_{1r,j}^{1_{1}}=p_{1r}^{1_{1}s_{\beta }}+d_{1r,j}^{,1\beta }+e_{1r,j}^{1_{1}s_{\beta }}$$

(6) $$\phi_{1r,j}^{1_{1}s_{\beta }}=\phi_{1r,j}^{s_{\beta }}-\phi_{1r,j}^{1_{1}}=p_{1r}^{1_{1}s_{\beta }}+\lambda_{j}(\delta_{1r,j}^{,1\beta }+N_{1r,j}^{1_{1}s_{\beta }})+\in_{1r,j}^{1_{1}s_{\beta }}$$

In classical double differencing for single satellite-type systems, such as GPS, the terms, $d_{1r,j}^{,1\beta }$ and $\delta_{1r,j}^{,1\beta }$ do not exist. However, as shown in Section 4.2, there may exist non-zero differential inter-satellite-type biases (ISTBs) for the BeiDou system if one uses mixed receiver types. That is, $\delta_{1r,j}^{,1\beta }\neq 0$ and $d_{1r,j}^{,1\beta }\neq 0$ for *β* ≠ 1.

The linearized DD observation equations corresponding to Equations (5) and (6) read:

(7) $$\text{E}(\Delta p_{1r,j}^{1_{1}s_{\beta }})={\text{g}}_{r}^{1_{1}s_{\beta }T}b+d_{1r,j}^{,1\beta },$$

(8) $$\text{E}(\Delta \phi_{1r,j}^{1_{1}s_{\beta }})={\text{g}}_{r}^{1_{1}s_{\beta }T}b+\lambda_{j}(\delta_{1r,j}^{,1\beta }+N_{1r,j}^{1_{1}s_{\beta }})$$

where E(·) denote the expectation operator, $\Delta p_{1r,j}^{1_{1}s_{\beta }}$ and $\Delta \phi_{1r,j}^{1_{1}s_{\beta }}$ are the observed-minus-computed code and phase observations, *b* is the baseline vector containing relative position components and $g_{r}^{1_{1}s_{\beta }}$ is the geometry vector given as $g_{r}^{1_{1}s_{\beta }}=u_{r}^{1_{1}}-u_{r}^{s_{\beta }}$, with $u_{r}^{s_{\beta }}$ the unit line-of-sight vector from receiver *r* to satellite *s_β_*.

The vectorial forms of the DD observation equations for the *j*th frequency read:

(9) $$\text{E}(y_{p;j})=G_{r}b+H_{\eta }h_{p;j}$$

(10) $$\text{E}(y_{\phi ;j})={\text{G}}_{r}b+\lambda_{j}z_{j}+\lambda_{j}H_{\eta }h_{\phi ;j}$$

with

(11) $$y_{p;j}={\left[\Delta p_{1r,j}^{1_{1}2_{1}},\ldots ,\Delta p_{1r,j}^{1_{1}m_{1}},\Delta p_{1r,j}^{1_{1}1_{2}},\ldots ,\Delta p_{1r,j}^{1_{1}m_{2}},\ldots ,\Delta p_{1r,j}^{1_{1}1_{\eta }},\ldots ,\Delta p_{1r,j}^{1_{1}m_{\eta }}\right]}^{T}$$

(12) $$y_{\phi ;j}={\left[\Delta \phi_{1r,j}^{1_{1}2_{1}},\ldots ,\Delta \phi_{1r,j}^{1_{1}m_{1}},\Delta \phi_{1r,j}^{1_{1}1_{2}},\ldots ,\Delta \phi_{1r,j}^{1_{1}m_{2}},\ldots ,\Delta \phi_{1r,j}^{1_{1}1_{\eta }},\ldots ,\Delta \phi_{1r,j}^{1_{1}m_{\eta }}\right]}^{T}$$

(13) $$G_{r}={\left[g_{r}^{1_{1}2_{1}},\ldots ,g_{r}^{1_{1}m_{1}},g_{r}^{1_{1}1_{2}},\ldots ,g_{r}^{1_{1}m_{2}},\ldots ,g_{r}^{1_{1}1_{\eta }},\ldots ,g_{r}^{1_{1}m_{\eta }}\right]}^{T}$$

(14) $$z_{j}={\left[N_{1r,j}^{1_{1}2_{1}},\ldots ,N_{1r,j}^{1_{1}m_{1}},N_{1r,j}^{1_{1}1_{2}},\ldots ,N_{1r,j}^{1_{1}m_{2}},\ldots ,N_{1r,j}^{1_{1}1_{\eta }},\ldots ,N_{1r,j}^{1_{1}m_{\eta }}\right]}^{T}$$

(15) $$h_{p;j}={\left[d_{1r,j}^{,12},\ldots ,d_{1r,j}^{,1\eta }\right]}^{T}$$

(16) $$h_{\phi ;j}={\left[\delta_{1r,j}^{,12},\ldots ,\delta_{1r,j}^{,1\eta }\right]}^{T}$$

and the (*m* − 1) × (*η* − 1) matrix:

(17) $$H_{\eta }=\left[\begin{array}{llll} 0 & 0 & \ldots & 0\\ e_{m_{2}} & 0 & \ldots & 0\\ 0 & e_{m_{3}} & \ldots & 0\\ \vdots & \vdots & \ddots & \vdots \\ 0 & 0 & \ldots & e_{m_{\eta }} \end{array}\right]$$

where *e_n_* is the *n*×1 vector of 1's.

When combining the single-epoch, multi-frequency linearized DD GNSS code and phase observation Equations (9) and (10), we obtain the mixed integer model of observation equations:

(18) $$\begin{array}{cc} \text{E}(y)=Az+Gb+Hh & \;z\in {\mathbb{Z}}^{f(m-1)},b\in {\mathbb{R}}^{3},h\in {\mathbb{R}}^{2f(\eta -1)} \end{array}$$

where $y={\left[y_{\phi }^{T},y_{p}^{T}\right]}^{T}$ is the 2*f* (*m* − 1) × 1 vector of linearized (observed-minus-computed) DD observations, with $y_{\phi }={\left[{y_{\phi ;1}}^{T},\ldots ,{y_{\phi ;f}}^{T}\right]}^{T}$, $y_{p}={\left[{y_{p;1}}^{T},\ldots ,{y_{p;f}}^{T}\right]}^{T}$, and $z={\left[{z_{,1}}^{T},\ldots ,{z_{,f}}^{T}\right]}^{T}$ is the *f* (*m* − 1) × 1 vector of unknown DD integer ambiguities, $h={\left[{h_{\phi }}^{T},\ldots ,{h_{p}}^{T}\right]}^{T}$ is the 2*f*(*η* − 1) × 1 vector of ISTBs with $h_{\phi }={\left[{h_{\phi ;1}}^{T},\ldots ,{h_{\phi ;f}}^{T}\right]}^{T}$ and $h_{p}={\left[{h_{p;1}}^{T},\ldots ,{h_{p;f}}^{T}\right]}^{T}$, *G* = *e*_2_⊗*e_f_*⊗*G_r_* is the 2*f*(*m*−1) × 3 geometry matrix, *A* = [*L^T^*, 0*^T^*]*^T^* is the 2*f*(*m* − 1) × *f*(*m*− 1) design matrix with *f*(*m* − 1) × *f*(*m* − 1) matrix *L* = diag(λ_1_,…, λ*_f_*) ⊗ *I_m_*_−1_, $H=\left[H_{\phi }^{T},H_{p}^{T}\right]$ is the 2*f*(*m* − 1) ×*f*(*η* − 1) design matrix with *f*(*m* − 1) × *f*(*η* − 1) matrix *H_Φ_* = diag(λ_1_,…, λ*_f_*) ⊗ *H_η_* and *f*(*m* − 1) ×*f*(*η* − 1) matrix *H_p_* = *I_f_* ⊗ *H_η_*, diag refers to the diagonal matrix formed by given arguments and ⊗ denotes the Kronecker product [51,52]. The model in Equation (18) contains a rank deficiency of *f(η* − 1), and it is not possible to simultaneously estimate the differential phase ISTB parameters and the double difference ambiguities of satellites that do not belong to the type of the pivot satellite. In the following, we describe four different processing strategies, namely ignoring ISTBs, removing rank deficiency, eliminating ISTBs and correcting for ISTBs.

### 2.1. Classical DD Model

The naive approach is to simply ignore the third term in Equation (18), resulting in the classical baseline model with a full-rank system:

(19) $$\begin{array}{cc} \text{E}(y)=Az+Gb & \;z\in {\mathbb{Z}}^{f(m-1)},b\in {\mathbb{R}}^{3} \end{array}$$

Hence, the redundancy of this classical model is equal to 2*f*(*m* − 1) − *f*(*m* − 1) − 3 = *f*(*m* − 1) − 3. As demonstrated in Section 4.3, ignoring the ISTBs results in catastrophic failure of ambiguity resolution.

### 2.2. Extended DD Model with Estimable ISTBs

The rank deficiency in Equation (18) can be removed by constraining a set of parameters (combinations) as the S-basis [53-55]. The number of S-basis constraints equals the size of the rank deficiency There are many possibilities to choose S-basis constraints. One such choice corresponds to a reparametrization in Equation (6), such that the DD ambiguities of the pivot satellites of second satellite types are combined with corresponding phase ISTBs:

(20) $$\begin{array}{cc} \phi_{1r,j}^{1_{1}1_{\beta }}=\rho_{1r}^{1_{1}1_{\beta }}+\lambda_{j}{\bar{\delta }}_{1r,j}^{,1\beta }+\varepsilon_{1r,j}^{1_{1}1_{\beta }}, & \;\text{for}\;\beta \neq 1 \end{array}$$

(21) $$\begin{array}{cc} \phi_{1r,j}^{1_{1}1_{\beta }}=\rho_{1r}^{1_{1}s_{\beta }}+\lambda_{j}\left({\bar{\delta }}_{1r,j}^{,1\beta }+N_{1r,j}^{1_{\beta }s_{\beta }}\right)+\varepsilon_{1r,j}^{1_{1}s_{\beta }}, & \;\text{for}\;\beta \neq 1,s_{\beta }\neq 1 \end{array}$$

where 1*_β_* is the pivot satellite of type-*β* satellites, ${\bar{\delta }}_{1r,j}^{,1\beta }=N_{1r,j}^{1_{1}1_{\beta }}+{\bar{\delta }}_{1r,j}^{,1\beta }$ and $N_{1r,j}^{1_{\beta }s_{\beta }}=N_{1r,j}^{1_{1}s_{\beta }}-N_{1r,j}^{1_{1}1_{\beta }}$. The estimable phase ISTB, ${\bar{\delta }}_{1r,j}^{,1\beta }$, is now biased by the inter-satellite-type ambiguity between the pivot satellites. Hence, to avoid the jumps due to the changes of reference satellites and cycle slips, we report the fractional part of the estimable phase ISTBs, which are sufficient for ISTB correction, as discussed in Section 2.4. Instead of lumping phase ISTB with the ambiguity of the first (pivot) satellite, one can lump the phase ISTB with the DD ambiguity of the satellite, other than the first satellite of second satellite type, and end up with different S-bases and different estimable phase ISTBs.

Another option, *i.e.*, another S-basis choice, is to lump the phase ISTB with the average of two or more DD ambiguities of the second satellite type. For example, if we lump the phase ISTB with the average of all DD ambiguities of the second satellite type, the DD phase observations of the second satellite type read:

(22) $$\begin{array}{cc} \phi_{1r,j}^{1_{1}1_{\beta }}=\rho_{1r}^{1_{1}s_{\beta }}-\frac{\lambda_{j}}{m_{\beta }}\sum_{s_{\beta }=2}^{m_{\beta }}N_{1r,j}^{{\bar{1}}_{\beta }s_{\beta }}+\lambda_{j}{\bar{\bar{\delta }}}_{1r,j}^{,1\beta }+\varepsilon_{1r,j}^{1_{1}s_{\beta }}, & \;\text{for}\;\beta \neq 1 \end{array}$$

(23) $$\begin{array}{cc} \phi_{1r,j}^{1_{1}s_{\beta }}=\rho_{1r}^{1_{1}s_{\beta }}+\frac{\lambda_{j}}{m_{\beta }}N_{1r,j}^{{\bar{1}}_{\beta }s_{\beta }}+\lambda_{j}{\bar{\bar{\delta }}}_{1r,j}^{,1\beta }+\varepsilon_{1r,j}^{1_{1}s_{\beta }} & \;\text{for}\;\beta \neq 1,s_{\beta }\neq 1 \end{array}$$

with estimable integer ambiguities:

(24) $$\begin{array}{cc} N_{1r,j}^{{\bar{1}}_{\beta }s_{\beta }}=\sum_{i_{\beta }=1}^{m_{\beta }}\left(N_{1r,j}^{1_{\beta }s_{\beta }}-N_{1r,j}^{1_{\beta }i_{\beta }}\right), & s_{\beta }=2,\ldots ,m_{\beta } \end{array}$$

and estimable phase ISTB:

(25) $${\bar{\bar{\delta }}}_{1r,j}^{,1\beta }=\delta_{1r,j}^{,1\beta }+\frac{1}{m_{\beta }}\sum_{s_{\beta }=1}^{m_{\beta }}N_{1r,j}^{1_{\beta }s_{\beta }}$$

For this choice of S-basis, however, the fractional part of the estimable phase ISTB is not necessarily equal to that of the actual phase ISTB. Having different choices, one should be cautious while interpreting or using the estimated phase ISTBs based on a given S-basis choice.

In this contribution, we consider the parametrization of Equations (20) and (21), which has the simple interpretation that the estimable integer ambiguities correspond to satellite type-specific double differencing (see Section 2.3), and moreover, it enables us to observe the fractional parts of the phase ISTBs (see Section 4.2). For our choice of S-basis, the full-rank (extended) model reads:

(26) $$\begin{array}{cc} \text{E}(y)=\bar{A}\bar{z}+Gb+H\bar{h} & \;\bar{z}\in {\mathbb{Z}}^{f(m-\eta )},b\in {\mathbb{R}}^{3},\bar{h}\in {\mathbb{R}}^{2f(\eta -1)} \end{array}$$

where $\bar{A}={\left[{\bar{L}}^{T},0^{T}\right]}^{T}$ is the *2f*(*m* − *1*) × *f*(*m* − *η*) design matrix with *f*(*m* − 1) × *f*(*m* − *η*) matrix *L* = diag(λ_1_,…, λ*_f_*) ⊗ blockdiag (I*_m_1__*_−1_, *C*_*m*_2__,…, *C_m_η__*), *C_n_* = [0 *I_n_*_−1_]*^T^* and blockdiag referring to the block diagonal matrix formed by given arguments, $\bar{z}={\left[{\bar{z}}_{,1}^{T},\ldots ,{\bar{z}}_{,f}^{T}\right]}^{T}$ is the *f*(*m* − *η*) × 1 vector of unknown DD integer ambiguities with ${\bar{z}}_{,j}={\left[{\bar{z}}_{,j}^{,1_{T}},\ldots ,{\bar{z}}_{,j}^{,\eta T}\right]}^{T}$ and ${\bar{z}}_{,j}^{,\beta }={\left[N_{1r,j}^{1_{\beta }2_{\beta }},\ldots ,N_{1r,j}^{1_{\beta }m_{\beta }}\right]}^{T}$, $\bar{h}={\left[{\bar{h}}_{\phi }^{T},\ldots ,h_{p}^{T}\right]}^{T}$ is the 2*f*(*η* − 1) × 1 vector of estimable ISTBs with ${\bar{h}}_{\phi }={\left[{\bar{h}}_{\phi ;1}^{T},\ldots ,{\bar{h}}_{\phi ;f}^{T}\right]}^{T}$ and ${\bar{h}}_{\phi ;j}=\left[{\bar{\delta }}_{1r,j}^{,12},\ldots ,{\bar{\delta }}_{1r,j}^{,1\eta }\right]$. The redundancy of this model is equal to 2*f*(*m*−1) −*f*(*m*−*η*) −2*f*(*η*−1) −3= *f*(*m* − *η*)−3.

### 2.3. Type-Specific DD Model

Since ISTBs are nuisance parameters, one can eliminate 2*f*(*η*− 1) ISTBs using the differencing operator, 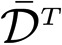 = *I*_2_ ⊗ *I_f_* ⊗ blockdiag $\left(I_{m_{1}-1},D_{m_{2}-1}^{T},\ldots ,D_{m_{\eta }-1}^{T}\right)$, in which $D_{n}^{T}=\left[-e_{n},I_{n}\right]$ is the differencing matrix. Pre-multiplying Equation (26) by 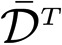 nullifies the third term and results in a type-specific DD model, which has a reference satellite (pivot) per satellite type and reads:

(27) $$\begin{array}{cc} \text{E}(y)=\bar{\bar{A}}\bar{z}+\bar{G}b & \bar{z}\in {\mathbb{Z}}^{f(m-\eta )},b\in {\mathbb{R}}^{3} \end{array}$$

where *ȳ*= 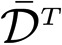 *y* the 2*f*(*m* − *η*) × 1 vector of type-specific DD observations, *A̿*= 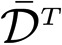
*Ā* = [*L̿^T^*, 0*^T^*]*^T^* is the 2*f*(*m* − *η*) × *f*(*m* − *η*) design matrix with *f*(*m* −*η*) × *f*(*m* − *η*) matrix *L̿* = diag(λ_1_,…, λ*_f_*) ⊗ blockdiag (*I*_*m*_1_−1_, *I_m_2__*_−1_,…, *I_m_η__*_−1_) and *Ḡ* = 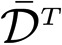
*G* is the 2*f*(*m* − *η*) × 3 geometry matrix. This model has 2*f*(*η* −1) less observations and 2*f*(*η* − 1) less unknown parameters than Equation (26). Hence, both models have the same redundancy and are equivalent.

### 2.4. ISTB-Corrected DD Model

As shown in Section 4.2, ISTBs are stable and can be assumed to be constant for a given receiver type pair. Hence, one can correct DD observations in Equation (18) with *a priori* estimates of ISTBs (calibration):

(28) $$\begin{array}{cc} \text{E}(\tilde{y})=Az+Gb & \;z\in {\mathbb{Z}}^{f(m-1)},b\in {\mathbb{R}}^{3} \end{array}$$

with *ỹ* = *ỹ* − *Hh̃* and *h̃* the vector of *a priori* ISTBs known through calibration and consisting of code and *fractional* phase ISTBs (see Section 4.2). Note that it is sufficient to use the fractional part of phase ISTB to correct phase observations, as the integer part of the phase ISTB can be lumped with the corresponding integer ambiguities without affecting integer ambiguity resolution. The redundancy of an ISTB-corrected system is equal to *f*(*m* − 1) − 3. Hence, this model is stronger than the extended model in Equation (26).

Finally, Table 1 summarizes the full-rank models described in the above. For further analyses in this paper, we only consider three models, namely, the classical DD model, ignoring ISTBs, the extended model and the classical DD model with ISTB correction. The type-specific DD model is equivalent to the extended DD model.

### 2.5. Stochastic Model

We assume elevation-dependent noise characteristics [56] for the undifferenced observables in Equations (1) and (2). That is, the standard deviation of the undifferenced observable, ς, can be written as:

(29) $$\sigma_{\varsigma }(\theta )=\sigma_{\varsigma_{0}}\left(1-a_{\varsigma_{0}}\exp\left(\frac{-\theta }{\theta_{\varsigma_{0}}}\right)\right)$$

where *θ* is the elevation angle of the corresponding satellite and *σ*_ς0_, a_ς0_ and *θ*_ς0_ are the elevation-dependent model parameters. We further assume that the receivers have similar characteristics and that the observation noise standard deviations can be decomposed as follows:

(30) $$\begin{array}{cc} \sigma_{\phi_{r,j}^{s_{\beta }}}= & \sigma_{r}\sigma_{\phi_{0}}\sigma_{,j}\iota^{s_{\beta }}\\ \sigma_{p_{r,j}^{s_{\beta }}}= & \sigma_{r}\sigma_{p_{0}}\sigma_{,j}\iota^{s_{\beta }}\\ \iota^{s_{\beta }}= & \left(1+a_{0}\exp\left(\frac{-\theta^{s_{\beta }}}{\theta_{0}}\right)\right) \end{array}$$

where *σ_r_* and *σ*_,*j*_ are the receiver, the frequency and the satellite-type-dependent weightings, respectively, and *σ_Φ_*_0_ and *σ_p_*_0_ are observation-dependent weightings.

To construct the stochastic model for the observations in Equation (18), consider the undifferenced observations reading:

(31) $$\zeta ={\left[\zeta_{1}^{T},\zeta_{2}^{T}\right]}^{T}$$

where $\zeta_{r}={\left[\phi_{r}^{T},p_{r}^{T}\right]}^{T}$, $\phi_{r}={\left[\phi_{r,1}\ldots \phi_{r,f}^{T}\right]}^{T}$, $\phi_{r,j}={\left[\phi_{r,j}^{1_{1}},\ldots ,\phi_{r,j}^{m_{1}},\ldots \phi_{r,j}^{1_{\eta }},\ldots ,\phi_{r,j}^{m_{\eta }}\right]}^{T}$, $p_{r}={\left[{p_{r,1}}^{T}\ldots {p_{r,f}}^{T}\right]}^{T}$, $p_{r,j}={\left[p_{r,j}^{1_{1}},\ldots ,p_{r,j}^{m_{1}},\ldots p_{r,j}^{1_{\eta }},\ldots ,p_{r,j}^{m_{\eta }}\right]}^{T}$ and $p_{r,j}^{s_{\beta }}$ and $\phi_{r,j}^{s_{\beta }}$ are the undifferenced code and phase observations defined in Equations (1) and (2). Using the noise characteristics of Equation (30) and assuming that the observables are normally distributed and mutually uncorrelated, the dispersion matrix of the observation vector, *ζ*, can be written as:

(32) $$\text{D}(\zeta )=Q_{r}⊗Q_{t}⊗Q_{f}⊗Q_{\theta }$$

where D(·) denotes the dispersion operator, $Q_{r}=\text{diag}\left[\sigma_{1}^{2},\sigma_{2}^{2}\right]$, $Q_{t}=\text{diag}\left[\sigma_{\phi_{0}}^{2},\sigma_{p_{0}}^{2}\right]$, $Q_{f}=\text{diag}\left[\sigma_{,1}^{2}\cdots \sigma_{,f}^{2}\right]$ are the co-factor matrices and $Q_{\theta }=\text{blockdiag}\left(Q_{\theta }^{,1},\ldots ,Q_{\theta }^{,\eta }\right)$ is the elevation-dependent weight matrix with $Q_{\theta }^{,\beta }=\text{diag}\left[{\left(\iota^{1_{\beta }}\right)}^{2},\ldots ,{\left(\iota^{m_{\beta }}\right)}^{2}\right]$. Using the DD operator, $D^{T}=D_{1}^{T}⊗I_{2}⊗I_{f}⊗D_{m-1}^{T}$, the dispersion matrix of the DD observations is then given as:

(33) $$\text{D}(y)=Q_{yy}=\text{D(}D^{T}\zeta \text{)}$$

(34) $$=(\sigma_{1}^{2}+\sigma_{2}^{2})Q_{t}⊗Q_{f}⊗(D_{m-1}^{T}Q_{\theta }\;D_{m-1})$$

For type-specific DD observations in Equation (27), the dispersion matrix is given as:

(35) $$\text{D}(\bar{y})=Q_{\bar{y}\bar{y}}={\bar{D}}^{T}Q_{yy}\bar{D}$$

(36) $$=(\sigma_{1}^{2}+\sigma_{2}^{2})Q_{t}⊗Q_{f}⊗\text{blockdiag}\;(D_{m_{1}-1}^{T}Q_{\theta }^{,1}\;D_{m_{1}-1},\ldots ,D_{m_{\eta }-1}^{T}Q_{\theta }^{,\eta }D_{m_{\eta }-1})$$

## 3. BeiDou Attitude Determination

Since attitude determination uses rigidly mounted antennas, the baseline length is **a priori** known and can be used to strengthen the underlying model. With the inclusion of the baseline length constraint to the models in Equations (19), (26) or (28) and with the stochastic model in Equation (34), we obtain the GNSS compass model [42,47]:

(37) $$\begin{array}{cc} \text{E}(y)=Az+Gb+Hh & \;\left\|b\right\|=l,z\in {\mathbb{Z}}^{\kappa },b\in {\mathbb{R}}^{3} \end{array}$$

(38) $$\text{D}(y)=Q_{yy}$$

where *l* is the known baseline length, ‖·‖ denotes the unweighted norm and *κ* is the number of integer ambiguities. The parameters for different models are defined as follows:
Classical model Equation (19):***y***= *y*,

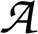
 = *A****z***= *z*,ℋ= [ ],***h*** = [ ],κ = *f*(*m*−1)Extended model Equation (26):***y*** = *y*,

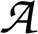
 = *Ā****z*** = *z̄*,ℋ =*H*,***h*** = *h̄*,κ = *f*(*m*− *η*)ISTBcorrected classical model Equation (28):***y*** = *ỹ*,

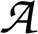
 = *A****z*** = *z*,ℋ = [ ],***h***= [ ],κ = *f*(*m*−1)

In the above, the baseline is constrained to lie on a sphere with radius *l* ( 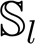 = {*b* ∈ ℝ^3^ | ‖b‖ = *l*}). Our objective is to solve for *b* in a least-squares sense, thereby taking the integer constraints on *z* and the quadratic constraint on vector *b* into account. Hence, the least-squares minimization problem that will be solved reads:

(39) $$\underset{z\in {\mathbb{Z}}^{\kappa },b\in {\text{S}}_{l}}{\min}{\left\|y-Az-Gb-Hh\right\|}_{Q_{yy}}^{2}$$

with ${\left\|\cdot \right\|}_{Q}^{2}={\left(\cdot \right)}^{T}Q^{-1}(\cdot )$. It is a quadratically-constrained (mixed) integer least-squares (QC-ILS) problem [41,47], for which no closed-form solution is available. In the following sections, we describe the method for solving Equation (39).

### 3.1. The Ambiguity Resolved Attitude

We now describe the steps for computing the integer ambiguity resolved attitude angles.

#### 3.1.1. The Real-Valued Float Solution

The float solution is defined as the solution of Equation (39) without the constraints. When we ignore the integer constraints on *z* and the quadratic constraint on *b*, the float solutions, *ẑ*, *b̂* and *ĥ*, and their variance-covariance matrices are obtained as follows:

(40) $${\left[\begin{array}{ccc} Q_{\hat{z}\hat{z}} & Q_{\hat{z}\hat{b}} & Q_{\hat{z}\hat{h}}\\ Q_{\hat{b}\hat{z}} & Q_{\hat{b}\hat{z}} & Q_{\hat{b}\hat{h}}\\ Q_{\hat{h}\hat{z}} & Q_{\hat{h}\hat{b}} & Q_{\hat{h}h} \end{array}\right]}^{-1}\cdot \left[\begin{array}{c} \hat{z}\\ \hat{b}\\ \hat{h} \end{array}\right]=\left[\begin{array}{c} A^{T}\\ G^{T}\\ H^{T} \end{array}\right]\;Q_{yy}^{-1}y$$

with:

(41) $$\left[\begin{array}{ccc} Q_{\hat{z}\hat{z}} & Q_{\hat{z}\hat{b}} & Q_{\hat{z}\hat{h}}\\ Q_{\hat{b}\hat{z}} & Q_{\hat{b}\hat{b}} & Q_{\hat{b}\hat{h}}\\ Q_{\hat{h}\hat{z}} & Q_{\hat{h}\hat{b}} & Q_{\hat{h}h} \end{array}\right]={\left(\left[\begin{array}{c} A^{T}\\ G^{T}\\ H^{T} \end{array}\right]\;Q_{yy}^{-1}\;\left[\text{A G H}\right]\right)}^{-1}$$

The *z*-constrained solution of *b* and its variance-covariance matrix can be obtained from the float solutions as follows:

(42) $$\hat{b}(z)=\hat{b}-Q_{\hat{b}\hat{z}}Q_{\hat{z}\hat{z}}^{-1}\;(\hat{z}-z)$$

(43) $$Q_{\hat{b}(z)\hat{b}(z)}=Q_{\hat{b}\hat{b}}-Q_{\hat{b}\hat{z}}Q_{\hat{z}\hat{z}}^{-1}Q_{\hat{z}\hat{b}}$$

The *z*- and *b*-constrained solution of *h* and its variance-covariance matrix can be obtained from the float solutions as follows:

(44) $$\hat{h}(z,b)=\hat{h}-[Q_{\hat{h}\hat{z}}Q_{\hat{h}\hat{b}}]\;{\left[\begin{array}{cc} Q_{\hat{z}\hat{z}} & Q_{\hat{z}\hat{b}}\\ Q_{\hat{b}\hat{z}} & Q_{\hat{b}\hat{b}} \end{array}\right]}^{-1}\;\left[\begin{array}{c} \hat{z}-z\\ \hat{b}-b \end{array}\right]$$

(45) $$Q_{\hat{h}(z,b)\hat{h}(z,b)}=Q_{\hat{h}\hat{h}}-[Q_{\hat{h}\hat{z}}Q_{\hat{h}\hat{b}}]\;{\left[\begin{array}{cc} Q_{\hat{z}\hat{z}} & Q_{\hat{z}\hat{b}}\\ Q_{\hat{b}\hat{z}} & Q_{\hat{b}\hat{b}} \end{array}\right]}^{-1}\;\left[\begin{array}{c} Q_{\hat{z}\hat{h}}\\ Q_{\hat{b}\hat{h}} \end{array}\right]$$

Using the above estimates, the original problem in Equation (39) can be decomposed as:

(46) $$\begin{array}{l} \underset{z\in {\mathbb{Z}}^{\kappa },b\in {\text{S}}_{l}}{\min}{\left\|y-Az-Gb-Hh\right\|}_{Q_{yy}}^{2}\\ ={\left\|\hat{e}\right\|}_{Q_{yy}}^{2}+\underset{z\in {\mathbb{Z}}^{\kappa }}{\min}\left({\left\|\hat{z}-z\right\|}_{Q_{\hat{z}\hat{z}}}^{2}+\underset{b\in {\text{S}}_{l}}{\min}\left[{\left\|\hat{b}(z)-b\right\|}_{Q_{\hat{b}(z)\hat{b}(z)}}^{2}+\underset{h}{\min}{\left\|\hat{h}(z,b)-h\right\|}_{Q_{\hat{h}(z,b)\hat{h}(z,b)}}^{2}\right]\right) \end{array}$$

with *ê* = *y*−*Aẑ* − *Gb̂* − *Hĥ* being the vector of least-squares residuals. Note that the first term on the right-hand side of Equation (46) does not depend on the unknown parameters, *z*, *b* and *h*, and is therefore constant. Unlike the third term, which is constrained, the last term can be reduced to zero for any given *z* and *b*.

#### 3.1.2. The Integer Ambiguity Resolution

Based on the orthogonal decomposition Equation (46), the quadratic-constrained integer minimiza-tion can be formulated as:

(47) $$\overset{⌣}{z}=\arg\underset{z\in {\mathbb{Z}}^{\kappa }}{\min}C(z)$$

with ambiguity objective function:

(48) $$C(z)={\left\|\hat{z}-z\right\|}_{Q_{\hat{z}\hat{z}}}^{2}+{\left\|\hat{b}(z)-\overset{⌣}{b}(z)\right\|}_{Q_{\hat{b}(z)\hat{b}(z)}}^{2}$$

where:

(49) $$\overset{⌣}{b}(z)=\arg\underset{b\in S_{l}}{\min}{\left\|\hat{b}(z)-b\right\|}_{Q_{\hat{b}(z)\hat{b}(z)}}^{2}$$

The cost function, *C*(*z*), is the sum of two coupled terms: the first weighs the distance from the float ambiguity vector, *ẑ*, to the nearest integer vector, *z*, in the metric of *Q_ẑẑ_*, while the second weighs the distance from the conditional float solution, *b̂*(*z*), to the nearest point on the sphere, 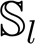, in the metric of *Q_b̂(z)b̂(z)_*.

Unlike with the standard LAMBDA method [29], the search space of the above minimization problem is non-ellipsoidal, due to the presence of the second term in the ambiguity objective function. Moreover, its solution requires the computation of a nonlinear-constrained least-squares problem (49) for every integer vector in the search space. In the C-LAMBDA method, this problem is mitigated through the use of easy-to-evaluate bounding functions [47]. Using these bounding functions, two strategies, namely the *Expansion* and the *Search and Shrink* strategies, were developed; see, e.g., [41,45]. These techniques avoid the computation of Equation (49) for every integer vector in the search space and compute the integer minimizer, *ẑ*, in an efficient manner.

#### 3.1.3. The Ambiguity Resolved Parameter Estimation

For a single baseline, *b* is related to the Euler-angles, *ξ* = [*Φ*, *θ*]*^T^*, with *Φ* the heading and *θ* the elevation, as *b*(*ξ*) = *lu*(*ξ*), where *u*(*ξ*) = [*c_θ_c_Φ_*, *c_θ_s_Φ_*, −*s_θ_*]*^T^*, with *s_α_* = sin(*α*) and *c_α_* = cos(*α*). The sought for attitude angles, *ξ* (*ž*), are the reparametrized solution of Equation (49). Using a first order approximation, the formal variance-covariance matrix of the ambiguity resolved, estimated heading and elevation angles are given by:

(50) $$Q_{\xi (\overset{⌣}{z})\xi (\overset{⌣}{z})}\approx \frac{1}{l^{2}}{\left(J_{u,\xi }{\left(\xi (\overset{⌣}{z})\right)}^{T}Q_{\hat{b}(z)\hat{b}(z)}^{-1}J_{u,\xi }(\xi (\overset{⌣}{z}))\right)}^{-1}$$

with Jacobian matrix:

(51) $$J_{u,\xi }(\xi )=\left[\begin{array}{cc} -s_{\phi }c_{\theta } & -c_{\phi }s_{\theta }\\ c_{\phi }c_{\theta } & -s_{\phi }s_{\theta }\\ 0 & -c_{\theta } \end{array}\right]$$

Finally, in the case of the extended model, ambiguity-corrected ISTB estimates, *ĥ*(*ž*,*b̌*(*ž*)), and the associated variance-covariance matrix, *Q_ĥ(z,b)ĥ(z,b)_* are obtained using Equations (44) and (45). The computation of the fractional phase ISTBs from these estimable ISTBs is discussed in Section 4.2.

## 4. Analyses

### 4.1. Measurement Campaign

**Figure 1.**
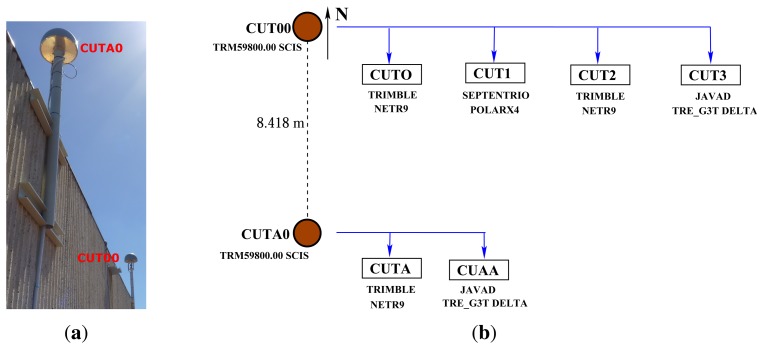
Curtin Global Navigation Satellite Systems (GNSS) antennas used for the real data campaign. (**a**) Antenna setup; (**b**) antenna geometry and receiver connectivity.

**Figure 2.**
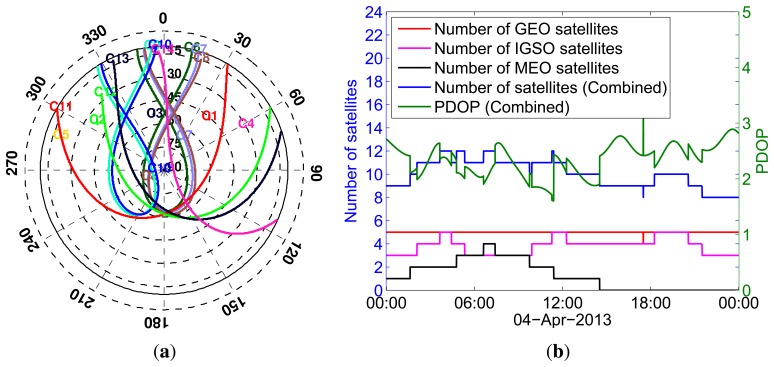
Satellite visibility of BeiDou satellites for Curtin Stations on April 4, 2013 with 10° elevation cut-off. (**a**) Skyplot; (**b**) number of satellites and position dilution of precision (PDOP).

The analyses in this paper are based on data sets from Curtin University's permanent GNSS stations and series of zero-baseline experiments. The first data set is from Curtin's permanent GNSS antennas (CUT00 and CUTA0) mounted on the roof of building 402 at the campus of Curtin University in Perth, Australia (Figure 1a). These antennas are connected to six BeiDou-enabled receivers, as summarised in Figure 1b, consisting of three Trimble NETR9, two Javad TRE_G3T DELTA and a Septentrio POLARx4 receivers. We considered the data from these receivers for five days from April 4 to 8, 2013. BeiDou satellite visibility for April 4 is shown in Figure 2. The data of various zero baselines for five days with a 30 s sampling interval is used to estimate ISTBs in Section 4.2, while the data of various short baselines between CUT00 and CUTA0 on April 7 with a 1 s sampling interval is used to analyse the impact of ISTBs on attitude determination in Section 4.3.

**Figure 3.**
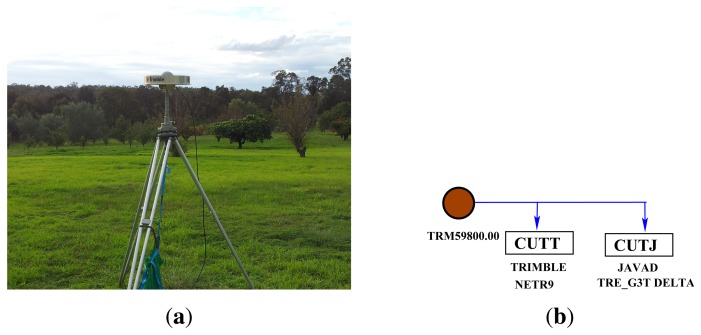
Zero-baseline experiments. (**a**) Antenna at Kalamunda experiment; (**b**) receiver-antenna connectivity.

**Figure 4.**
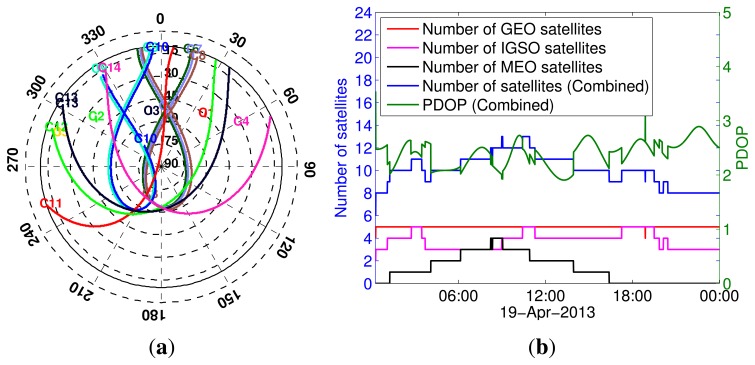
Satellite visibility of BeiDou satellites for open space experiment on April 19, 2013 with 10° elevation cut-off. (**a**) Skyplot; (**b**) number of satellites and PDOP.

In addition to the data from Curtin's permanent stations, we also carried out a series of zero-baseline experiments: two in an open space in Curtin University and another two in an open space in Kalamunda, Western Australia (about 17 km from Curtin University), each for three consecutive days (Table 2). As shown in Figure 3b, a single antenna (Figure 3a) was connected to two BeiDou-enabled receivers (Trimble NETR9 and Javad Javad TRE G3T DELTA). Figure 4 shows the visibility of BeiDou satellites on April 19, 2013. These zero-baseline data sets (with a 30 s sampling interval) are also used to estimate and validate ISTBs in Section 4.2. The stochastic model parameters of the elevation-dependent model Equation (29) for the receivers are reported in Table 3. Since the receivers, except Trimble NETR9, track only B1 and B2 signals, single- and dual-frequency analyses are considered in the paper.

### 4.2. BeiDou Inter-Satellite-Type Bias (ISTB)

First, we considered the estimation of differential ISTBs using zero baseline data. Since the geometry term vanishes for a zero baseline problem, the estimation of code and phase ISTBs for each frequency are decoupled. Using the extended model in Equation (26) and the associated stochastic model in Equation (34), the decoupled system for the differential code ISTBs at the jth frequency is given as:

(52) $$\begin{array}{cc} \text{E}(y_{p;j})=H_{\eta }h_{p;j}, & h_{p;j}\in {\mathbb{R}}^{\eta -1} \end{array}$$

(53) $$\text{D}(y_{p;j})=Q_{y_{p;j}y_{p;j}}=(\sigma_{1}^{2}+\sigma_{r}^{2})\sigma_{p_{0}}^{2}\sigma_{,j}^{2}\;(D_{m-1}^{T}Q_{\theta }\;D_{m-1})$$

The estimates of differential code ISTBs are then given by the least-squares solution of the above system. Similarly, using the extended model in Equation (26) and the associated stochastic model in Equation (34), the decoupled system for the differential estimable phase ISTBs at the *j*th frequency is given as:

(54) $$\text{E}(y_{\phi ;j})={\bar{L}}_{,j}{\bar{z}}_{,j}+\lambda_{j}H_{\eta }{\bar{h}}_{\phi ;j},\;{\bar{z}}_{,j}\in {\mathbb{Z}}^{m-\eta },\;{\bar{h}}_{\phi ;j}\in {\mathbb{R}}^{\eta -1}$$

(55) $$\text{D}(y_{\phi ;j})=Q_{y_{\phi ;j}y_{\phi ;j}}=(\sigma_{1}^{2}+\sigma_{r}^{2})\sigma_{\phi_{0}}^{2}\sigma_{,j}^{2}\;(D_{m-1}^{T}Q_{\theta }D_{m-1})$$

with *L̂,_j_* = λ*_j_*blockdiag (*I*_m_1_−1_, *C*_*m*_2__,…, *C_m_η__*). First, the float solution of the above full-rank square system is obtained by ignoring integer constraints:

(56) $$\left[\begin{array}{c} {\hat{\bar{z}}}_{,j}\\ {\hat{\bar{h}}}_{\phi ;j} \end{array}\right]={\left[{\bar{L}}_{,j},\lambda_{j}H_{\eta }\right]}^{-1}y_{\phi ;j}$$

(57) $$\left[\begin{array}{cc} Q_{{\hat{\bar{z}}}_{,j}{\hat{\bar{z}}}_{,j}} & Q_{{\hat{\bar{z}}}_{,j}{\hat{\bar{h}}}_{\phi ;j}}\\ Q_{{\hat{\bar{h}}}_{\phi ;j}{\hat{\bar{z}}}_{,j}} & Q_{{\hat{\bar{h}}}_{\phi ;j}{\hat{\bar{h}}}_{\phi ;j}} \end{array}\right]={\left[{\bar{L}}_{,j},\lambda_{j}H_{\eta }\right]}^{-1}Q_{y_{\phi ;j}y_{\phi ;j}}{\left[\begin{array}{c} {\bar{L}}_{,j}\\ \lambda_{j}H_{\eta } \end{array}\right]}^{-1}$$

Since the above system is driven by phase measurement noise, simple rounding of *z̄̂*_,*j*_ yields integer ambiguities, (inline) The estimates for the estimable phase ISTBs are then given by:

(58) $${\hat{\bar{h}}}_{\phi ;j}({\overset{⌣}{\bar{z}}}_{,j})={\hat{\bar{h}}}_{\phi ;j}-Q_{{\hat{\bar{h}}}_{\phi ;j}{\hat{\bar{z}}}_{,j}}Q_{{\hat{\bar{z}}}_{,j}{\hat{\bar{z}}}_{,j}}^{-1}\left({\hat{\bar{z}}}_{,j}-{\overset{⌣}{\bar{z}}}_{,j}\right)$$

(59) $$Q_{{\hat{\bar{h}}}_{\phi ;j}({\hat{\bar{z}}}_{,j}){\hat{\bar{h}}}_{\phi ;j}({\overset{⌣}{\bar{z}}}_{,j})}=Q_{{\hat{\bar{h}}}_{\phi ;j}{\hat{\bar{h}}}_{\phi ;j}}-Q_{{\hat{\bar{h}}}_{\phi ;j}{\hat{\bar{z}}}_{,j}}Q_{{\hat{\bar{z}}}_{,j}{\hat{\bar{z}}}_{,j}}^{-1}Q_{{\hat{\bar{z}}}_{,j}{\hat{\bar{h}}}_{\phi ;j}}$$

Since these estimable phase ISTBs are the sum of actual phase ISTBs and corresponding ambiguities of reference satellites of the second satellite type, the estimates are affected by integer jumps, due to the cycle slips and the changes of reference satellites. Hence, we report only fractional phase ISTBs (the residuals of integer rounding) that are sufficient for ISTB correction as discussed in Section 2.4. That is, the fractional phase ISTBs, ${\hat{\tilde{h}}}_{\phi ;j}({\overset{⌣}{\bar{z}}}_{,j})={\hat{\bar{h}}}_{\phi ;j}({\overset{⌣}{\bar{z}}}_{,j})-\text{round}\left({\hat{\bar{h}}}_{\phi ;j}({\overset{⌣}{\bar{z}}}_{,j})\right)$, where ‘round’ refers to the closest integer to the given estimate. However, these fractional phase ISTB estimates are ambiguous if they are equal to or close to a half cycle (e.g., for a half cycle, simple rounding will yield either +0.5 − *ϵ* or −0.5 + *ϵ*, depending on the noise, *ϵ*). For this situation, we use either “floor” (resulting in the residual for the nearest integer that is smaller than the given estimate) or “ceiling” (resulting in the residual for the nearest integer that is larger than the given estimate; with the sign convention in Table 4. Note that one is free to choose any sign convention, as long as it preserves the consistency of the double difference ambiguities when the reference receiver and/or the reference satellite type changes.

**Figure 5.**
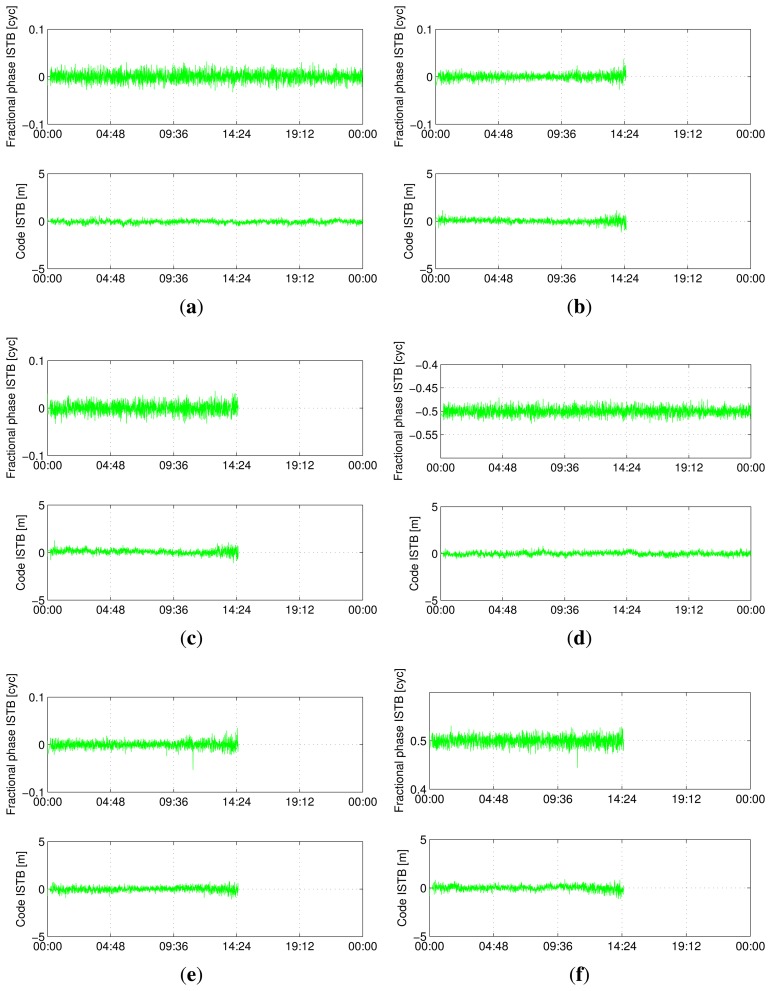
Estimated BeiDou differential ISTB for CUT0-CUT1 on 4 April 2013 (Trimble-Septentrio, zero-baseline). (**a**) B1 ISTB for IGSO-GEO; (**b**) B1 ISTB for IGSO-MEO; (**c**) B1 ISTB for GEO-MEO; (**d**) B2 ISTB for IGSO-GEO; (**e**) B2 ISTB for IGSO-MEO; (**f**) B2 ISTB for GEO-MEO.

**Figure 6.**
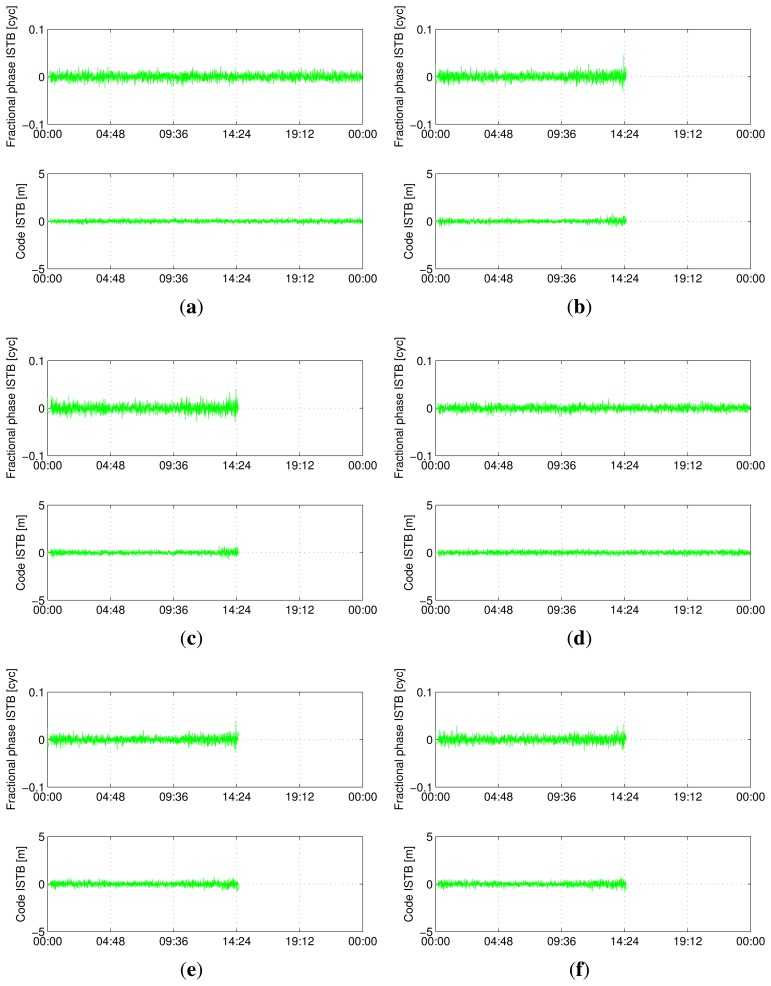
Estimated BeiDou differential ISTB for CUT0-CUT2 on 4 April 2013 (Trimble-Trimble, zero-baseline). (**a**) B1 ISTB for IGSO-GEO; (**b**) B1 ISTB for IGSO-MEO; (**c**) B1 ISTB for GEO-MEO; (**d**) B2 ISTB for IGSO-GEO; (**e**) B2 ISTB for IGSO-MEO; (**f**) B2 ISTB for GEO-MEO.

**Figure 7.**
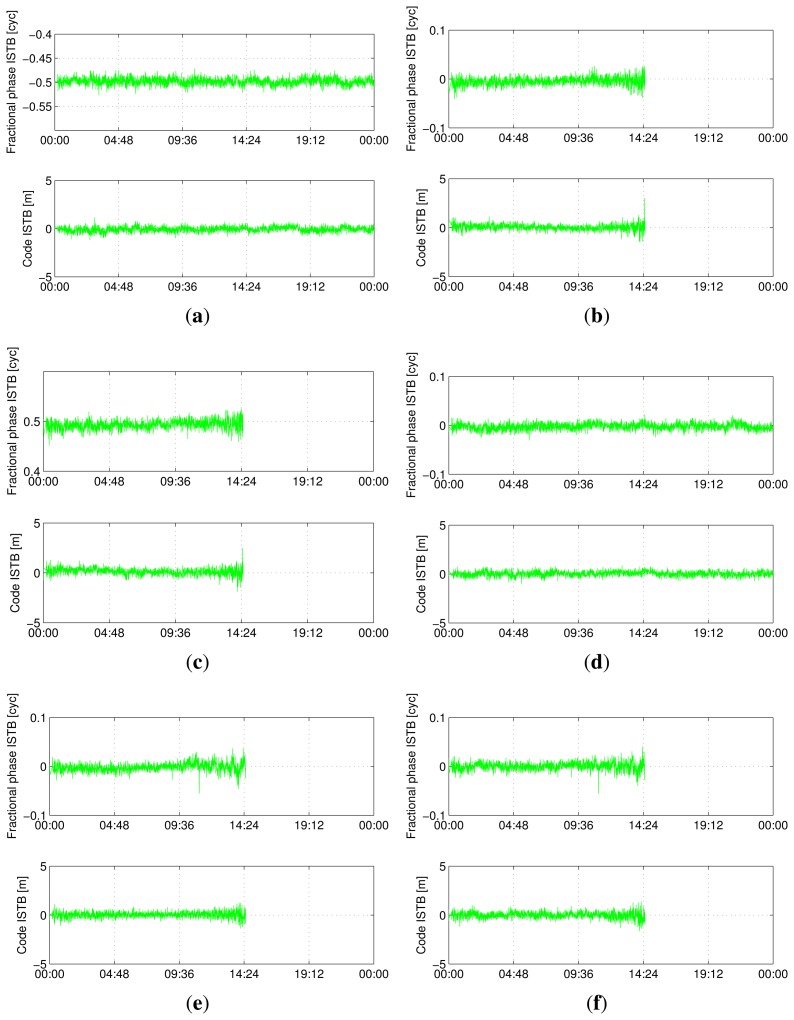
Estimated BeiDou differential ISTB for CUT0-CUT3 on 4 April 2013 (Trimble-Javad, zero-baseline). (**a**) B1 ISTB for IGSO-GEO; (**b**) B1 ISTB for IGSO-MEO; (**c**) B1 ISTB for GEO-MEO; (**d**) B2 ISTB for IGSO-GEO; (**e**) B2 ISTB for IGSO-MEO; (**f**) B2 ISTB for GEO-MEO.

**Figure 8.**
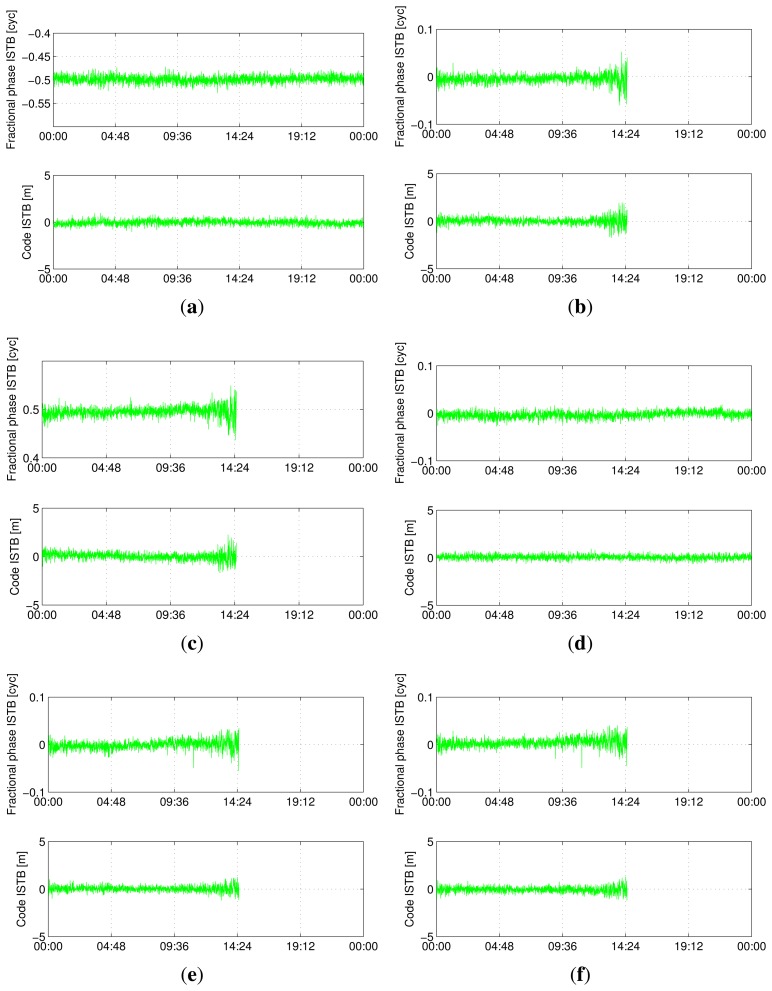
Estimated BeiDou differential ISTB for CUTA-CUAA on 4 April 2013 (Trimble-Javad, zero-baseline). (**a**) B1 ISTB for IGSO-GEO; (**b**) B1 ISTB for IGSO-MEO; (**c**) B1 ISTB for GEO-MEO; (**d**) B2 ISTB for IGSO-GEO; (**e**) B2 ISTB for IGSO-MEO; (**f**) B2 ISTB for GEO-MEO.

**Figure 9.**
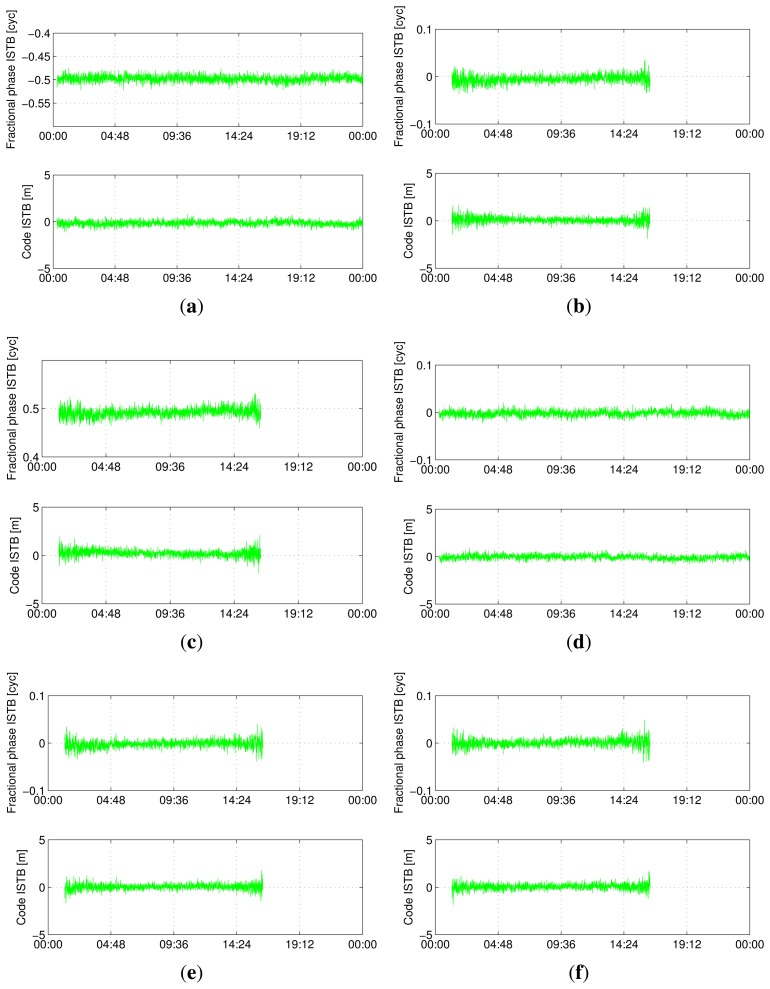
Estimated BeiDou differential ISTB for CUTT-CUTJ on 19 April 2013 at Curtin open space (Trimble-Javad, zero-baseline). (**a**) B1 ISTB for IGSO-GEO; (**b**) B1 ISTB for IGSO-MEO; (**c**) B1 ISTB for GEO-MEO; (**d**) B2 ISTB for IGSO-GEO; (**e**) B2 ISTB for IGSO-MEO; (**f**) B2 ISTB for GEO-MEO.

Figure 5, 6, 7 and 8 show the time series of ISTB estimates for zero-baselines formed by Curtin receivers (CUT0-CUT1, CUT0-CUT2, CUT0-CUT3 and CUTA-CUAA) on April 4, 2013. The first two columns are for ISTBs of GEO and MEO satellite types with an IGSO satellite as the reference, while the last column is for MEO ISTBs with respect to GEO satellites. Similar results for Curtin's open space experiment are given in Figure 9, matching with the results of the same receiver pair (Trimble-Javad) in Figure 7 and 8. The gaps in the MEO-related time series are due to the unavailability of visible MEO satellites during those periods.

It is observed that the estimated ISTBs (monitored for several days) are very stable and can be used to calibrate BeiDou observations. In the following, we compute the ISTB corrections using epoch-by-epoch estimates of several days. Let us consider the time series of the ith ITSB and associated standard deviations, ${\left\{h_{i;k},\sigma_{h_{i;k}}\right\}}_{\kappa =1}^{K}$ where *K* is the number of epochs and *h_i:k_* is the estimated code or phase ISTB at time *k*. Assuming these estimates are uncorrelated over time, we formulate the following least-squares problem for the bias estimation:

(60) $$\text{E}(h_{i})=e_{K}u_{i}$$

(61) $$\text{D}(h_{i})=Q_{h_{i}h_{i}}=\text{diag}\left[\sigma_{h_{i:1}},\ldots ,\sigma_{h_{i:K}}\right]$$

with *h_i_* = [*h_i_*_:1_,…, *h_i:K_*]*^T^* the *K* × 1 vector of epoch-by-epoch ISTB estimates for the ith ISTB. The least-squares estimate (weighted mean) of the bias and its standard deviation are given as:

(62) $${\hat{u}}_{i}=\frac{\sum_{k=1}^{K}h_{i:k}/\sigma_{h_{i:k}}^{2}}{\sum_{k=1}^{K}1/\sigma_{h_{i:k}}^{2}}$$

(63) $$\sigma_{{\hat{u}}_{i}}=\frac{1}{\sqrt{\sum_{k=1}^{K}1/\sigma_{h_{i:k}}^{2}}}$$

Table 5 and 6 summarize the ISTB estimates and their standard deviations, clearly indicating the existence of non-zero ISTBs (highlighted using bold text) between dissimilar receiver types. It was observed that the estimated ISTBs are constant for given receiver-antenna connectivity and receiver operating environment. Nevertheless, it was found that the observed code ISTBs do not significantly affect ambiguity resolution and the consequent ambiguity resolved phase-only baseline estimation. However, the phase ISTBs severely affect ambiguity resolution, especially in the case of half-cycle phase ISTBs. As summarized in Table 7, GEO satellites have phase ISTBs of half-cycles with respect to IGSO/MEO satellites in the case of mixed receivers. Note that phase ISTBs for other receiver pairs can be deduced from estimates in Table 7. For example, with the sign convention in Table 4, the Septentrio-Javad pair has phase ISTBs of -0.5 cycle and 0.5 cycle for B1 and B2, respectively. Note that, for attitude determination with the ISTB-corrected model in the following section, we only correct phase observations with half cycles, as other (code) biases were found to be small enough to not affect the ambiguity resolution significantly.

### 4.3. Effect of ISTBs on Attitude Determination

Next, we analysed the impact of ISTBs on BeiDou single- and dual-frequency instantaneous attitude determination using the standard LAMBDA and C-LAMBDA methods comparing three processing approaches, namely, the classical DD model with ignoring ISTBs in Equation (19), the extended model in Equation (26) and the classical DD model with ISTB correction in Equation (28). Results for different receiver pairs (CUT0-CUTA, CUT0-CUAA, CUT1-CUTA and CUT1-CUAA), which consist of mixed receivers forming a short baseline of 8.418 m, as shown in Figure 1b, are discussed in the following. Note that these receiver pairs are not used in the computation of ISTBs in Section 4.2. We considered two performance measures for our analyses; the first one is the empirical instantaneous ambiguity success fraction (relative frequency), which is defined as:

(64) $$\text{success fraction}=\frac{\text{number of correctly fixed epochs}}{\text{total number of epochs}}$$

where the true ambiguities are computed using known antenna coordinates in WGS84, as the antennas used are part of Curtin's permanent stations. However, only length information is used for C-LAMBDA processing. The second performance measure is the ambiguity fixed angular estimation accuracy, which is given by the formal and empirical standard deviations of attitude angular estimates.

Table 8, 9 and 10 report the instantaneous ambiguity success fraction for single-frequency B1, single-frequency B2 and dual frequency B1-B2 processing, respectively. The first row in each table corresponds to the baseline with the same receiver type for which ISTBs are zero and correction is not needed. The third row in Table 8 and the second row in Table 9 correspond to baselines with dissimilar receiver types, which have zero ISTBs for corresponding frequencies. The benefits of using C-LAMBDA, which makes use of known baseline length, are highlighted using bold text. Furthermore, catastrophic failures of ambiguity resolution by ignoring non-zero ISTBs are highlighted with emphasized text. Hence, it is wise to use the extended model (or the type-specific DD model) if one does not have the knowledge of ISTB between dissimilar receiver pairs. However, the best processing strategy is to use ISTB-corrected classical double differencing. With ISTB calibration, the C-LAMBDA method yields single-frequency instantaneous attitude determination.

Finally, Table 11 reports ambiguity fixed angular accuracy for single- and dual-frequency processing. Since the baselines (8.418 m) considered in these analyses are formed by receivers with similar noise characteristics, the *ambiguity fixed* angular standard deviations are the same for all cases, except the cases with catastrophic failure of ambiguity resolution. Hence, we report the average angular standard deviation of all other cases. Single-frequency processing with either B1 or B2 yields the same angular accuracy. The improved dual-frequency angular accuracy reflects the increased redundancy.

## 5. Summary and Conclusions

In this contribution, we investigated the existence of BeiDou inter-satellite-type biases (ISTBs) and their impact on standalone BeiDou attitude determination with mixed receiver types. We considered an extended GNSS double difference model incorporating all possible differential ISTBs among the three BeiDou satellite types (GEO, IGSO and MEO), together with three processing approaches, namely, one based on the classical double differenced model, ignoring the ISTBs, another based on the extended double differenced model, incorporating the ISTBs, and a third one based on the ISTB-corrected classical double differenced model. Our analyses using two real data sets with three different receiver types demonstrate the existence of non-zero ISTBs between different satellite types. The estimated ISTBs are stable and can be used to correct mixed receiver BeiDou attitude determination. It was observed that the estimated ISTBs are constant for a given receiver-antenna connectivity and receiver operating environment. Nevertheless, it was found that the observed code ISTBs do not significantly affect ambiguity resolution and the consequent ambiguity resolved phase-only baseline estimation. However, the mixed receiver half-cycle phase ISTBs severely affect ambiguity resolution. This finding is an important warning for mixed receiver type users, including precise point positioning users [54,55,57,58]. Moreover, it may also trigger GNSS receiver manufacturers to develop mutually consistent measurement extractions, as they are in the early stage of BeiDou-enabled receiver developments. Furthermore, it is suggested to use the extended model or, equivalently, the type-specific DD model, if one does not have the knowledge of ISTBs between dissimilar receiver pairs. However, the best processing strategy is to use the ISTB-corrected classical double differencing procedure. With ISTB correction, the C-LAMBDA method enables single-frequency, instantaneous attitude determination capability in the Asia-Pacific region with the current BeiDou constellation.

## Figures and Tables

**Table 1.** Redundancies of models considered. DD, double differencing; ISTB, inter-satellite-type bias.

**Model**	**Redundancy**
Classical DD with ignoring ISTBs Equation (19)	*f*(*m* − 1) −3
Extended model (26)	*f*(*m* − *η*) − 3
Type-specific DD model (27)	*f*(*m* − *η*) − 3
Classical DD with ISTB correction Equation (28)	*f*(*m* − 1) − 3

**Table 2.** Zero-baseline experiments with Trimble NETR9 and Javad TRE G3T DELTA receivers.

**Experiments**	**Duration**
Curtin 1	April 19–21
Curtin 2	April 29–May 01
Kalamunda 1	May 19–21
Kalamunda 2	May 29–31

**Table 3.** Elevation-dependent stochastic model parameters Equation (29) used in the real data campaigns.

**Frequency**	**Code**			**Phase**		
	*σ_p_*_o_ [cm]	*a_p_*_o_	*ϵ_p_*_o_ [deg]	σ*_Φ_*_o_ [mm]	*a_Φ_*_o_	*ϵ_Φ_*_o_ [deg]
B1 and B2	20	5	15	2	5	15

**Table 4.** Sign convention for fractional phase ISTBs that are equal to or close to a half cycle, with Trimble as the reference receiver. GEO, Geostationary Earth Orbit; IGSO, Inclined Geosynchronous Satellite Orbit; MEO, Medium Earth Orbit.

**Satellite type pairs**	**Rounding method/Sign**
GEO-IGSO	floor/+
GEO-MEO	floor/+
IGSO-GEO	ceiling/−
IGSO-MEO	ceiling/−
MEO-GEO	ceiling/+
MEO-IGSO	floor/−

**Table 5.** Estimated ISTBs using five days of data from Curtin's zero baselines.

**Receiver Pair**	**Frequency**	**IGSO-GEO**		**IGSO-MEO**		**GEO-MEO**	
**Phase ISTB/std (cyc)**	**Code ISTB/std (m)**	**Phase ISTB/std (cyc )**	**Code ISTB/std (m)**	**Phase ISTB/std (cyc)**	**Code ISTB/std (m)**
CUT0-CUT1	B1	0.00/ 0.000	−**0.07**/ 0.002	0.00/ 0.000	**0.03**/ 0.003	0.00/ 0.000	**0.10**/ 0.003
(Trimble-Septentrio)	B2	−**0.50**/ 0.000	−**0.01**/ 0.002	0.00/ 0.000	**0.01**/ 0.003	0.50/ 0.000	0.02/ 0.003

CUT0-CUT2	B1	0.00/ 0.000	0.00/ 0.002	0.00/ 0.000	0.00/ 0.003	0.00/ 0.000	0.00/ 0.003
(Trimble-Trimble)	B2	0.00/ 0.000	0.00/ 0.002	0.00/ 0.000	0.00/ 0.003	0.00/ 0.000	0.00/ 0.003

CUT0-CUT3	B1	−**0.50**/ 0.000	−**0.06**/ 0.002	0.00/ 0.000	**0.04**/ 0.003	0**.49**/ 0.000	**0.10**/ 0.003
(Trimble-Javad)	B2	−0.00/ 0.000	**0.03**/ 0.002	0.00/ 0.000	**0.05**/ 0.003	0.00/ 0.000	**0.01**/ 0.004

CUTA-CUAA	B1	−**0.50**/ 0.000	−**0.06**/ 0.002	0.00/ 0.000	**0.01**/ 0.003	**0.50**/ 0.000	**0.07**/ 0.003
(Trimble-Javad)	B2	0.00/ 0.000	**0.04**/ 0.002	0.00/ 0.000	**0.03**/ 0.003	0.00/ 0.000	−**0.02**/ 0.003

**Table 6.** Estimated ISTBs using data from zero baseline experiments with Trimble-Javad receiver pairs: two in Curtin University and another two in Kalamunda (Table 2).

**Experiment**	**Frequency**	**IGSO-GEO**		**IGSO-MEO**		**GEO-MEO**	
		**Phase ISTB/std(cyc)**	**Code ISTB/std (m)**	**Phase ISTB/std(cyc)**	**Code ISTB/std (m)**	**Phase ISTB/std(cyc)**	**Code ISTB/std (m)**
Curtin 1	B1	−**0.50**/0.000	−**0.17**/0.003	0.00/0.000	**0.06**/0.005	**0.49**/0.000	**0.23**/0.005
	B2	0.00/0.000	−**0.06**/0.003	0.00/0.000	**0.04**/0.005	0.00/0.000	**0.10**/0.005

Curtin 2	B1	−**0.50**/0.000	−**0.16**/0.003	0.00/0.000	**0.05**/0.006	**0.49**/0.000	**0.20**/0.006
	B2	0.00/0.000	−**0.06**/0.003	0.00/0.000	**0.03**/0.006	0.00/0.000	**0.08**/0.006

Kalamunda 1	B1	−**0.50**/0.000	−**0.12**/0.003	0.00/0.000	**0.04**/0.005	**0.49**/0.000	**0.16**/0.005
	B2	0.00/0.000	−**0.03**/0.003	0.00/0.000	**0.03**/0.005	0.00/0.000	**0.06**/0.005

Kalamunda 2	B1	−**0.50**/0.000	−**0.11**/0.003	0.00/0.000	**0.04**/0.005	**0.49**/0.000	**0.16**/0.005
	B2	0.00/0.000	−**0.02**/0.003	0.00/0.000	**0.04**/0.005	0.00/0.000	**0.07**/0.005

**Table 7.** Differential phase ISTB between GEO and IGSO/MEO satellites with Trimble as the pivot receiver (cyc) and based on the sign convention considered in Table 4

**Frequency**	**Trimble**	**Septentrio**	**Javad**
B1	0	0	−0.5
B2	0	−0.5	0

**Table 8.** Instantaneous ambiguity success fractions (relative frequencies) for single-frequency (B1) processing.

**Baseline**	**Classical DD model Equation (19)**		**Extended model Equation (26)**		**Classical DD model with ISTB correction Equation (28)**	
	**LAMBDA**	**C-LAMBDA**	**LAMBDA**	**C-LAMBDA**	**LAMBDA**	**C-LAMBDA**
CUT0-CUTA (Trimble-Trimble)	0.98	**1.00**	0.77	0.99	0.98	**1.00**
CUT0-CUAA (Trimble-Javad)	*0.00*	*0.00*	0.75	0.99	0.97	**1.00**
CUT1-CUTA (Septentrio-Trimble)	0.99	**1.00**	0.87	**1.00**	0.99	**1.00**
CUT1-CUAA (Septentrio-Javad)	*0.00*	*0.00*	0.86	**1.00**	0.99	**1.00**

**Table 9.** Instantaneous ambiguity success fractions (relative frequencies) for single-frequency (B2) processing.

**Baseline**	**Classical DD model Equation (19)**		**Extended model Equation (26)**		**Classical DD model with ISTB correction Equation (28)**	
	**LAMBDA**	**C-LAMBDA**	**LAMBDA**	**C-LAMBDA**	**LAMBDA**	**C-LAMBDA**
CUT0-CUTA (Trimble-Trimble)	0.98	**1.00**	0.86	**1.00**	0.98	**1.00**
CUT0-CUAA (Trimble-Javad)	0.99	**1.00**	0.88	**1.00**	0.99	**1.00**
CUT1-CUTA (Septentrio-Trimble)	*0.00*	*0.00*	0.94	**1.00**	0.99	**1.00**
CUT1-CUAA (Septentrio-Javad)	*0.00*	*0.00*	0.96	1.00	1.00	1.00

**Table 10.** Instantaneous ambiguity success fractions (relative frequencies) for dual-frequency (B1–B2) processing.

**Baseline**	**Classical DD model Equation (19)**		**Extended model Equation (26)**		**Classical DD model with ISTB correction Equation (28)**	
	**LAMBDA**	**C-LAMBDA**	**LAMBDA**	**C-LAMBDA**	**LAMBDA**	**C-LAMBDA**
CUT0-CUTA (Trimble-Trimble)	1.00	1.00	1.00	1.00	1.00	1.00
CUT0-CUAA (Trimble-Javad)	*0.00*	*0.00*	1.00	1.00	1.00	1.00
CUT1-CUTA (Septentrio-Trimble)	*0.00*	*0.00*	1.00	1.00	1.00	1.00
CUT1-CUAA (Septentrio-Javad)	*0.00*	*0.00*	1.00	1.00	1.00	1.00

**Table 11.** Empirical and formal (given in brackets) angular standard deviation (deg).

	**Single-Frequency (B1)**	**Single-Frequency (B2)**	**Dual-Frequency (B1–B2)**
Heading	0.02 (0.02)	0.02 (0.02)	0.01 (0.01)
Elevation	0.04 (0.04)	0.04 (0.04)	0.03 (0.03)
